# Adhesive properties of *Enterobacter sakazakii *to human epithelial and brain microvascular endothelial cells

**DOI:** 10.1186/1471-2180-6-58

**Published:** 2006-06-26

**Authors:** Jean-Philippe Mange, Roger Stephan, Nicole Borel, Peter Wild, Kwang Sik Kim, Andreas Pospischil, Angelika Lehner

**Affiliations:** 1 Institute for Food Safety and Hygiene, Vetsuisse Faculty, University of Zurich, CH-8057 Zurich, Switzerland; 2 Institute of Veterinary Pathology, Vetsuisse Faculty, University of Zurich, CH-8057 Zurich, Switzerland; 3 Institute for Veterinary Anatomy, Vetsuisse Faculty, University of Zurich, CH-8057 Zurich, Switzerland; 4 Pediatric Infectious Diseases, School of Medicine, John Hopkins University, Baltimore, MD 21287, USA

## Abstract

**Background:**

*Enterobacter sakazakii *is an opportunistic pathogen that has been associated with sporadic cases and outbreaks causing meningitis, necrotizing enterocolitis and sepsis especially in neonates. However, up to now little is known about the mechanisms of pathogenicity in *E. sakazakii*. A necessary state in the successful colonization, establishment and ultimately production of disease by microbial pathogens is the ability to adhere to host surfaces such as mucous membranes, gastric and intestinal epithelial or endothelial tissue.

This study examined for the first time the adherence ability of 50 *E. sakazakii *strains to the two epithelial cell lines HEp-2 and Caco-2, as well as the brain microvascular endothelial cell line HBMEC. Furthermore, the effects of bacterial culture conditions on the adherence behaviour were investigated. An attempt was made to characterize the factors involved in adherence.

**Results:**

Two distinctive adherence patterns, a diffuse adhesion and the formation of localized clusters of bacteria on the cell surface could be distinguished on all three cell lines. In some strains, a mixture of both patterns was observed. Adherence was maximal during late exponential phase, and increased with higher MOI. The adhesion capacity of *E. sakazakii *to HBMEC cells was affected by the addition of blood to the bacteria growth medium. Mannose, hemagglutination, trypsin digestion experiments and transmission electron microscopy suggested that the adhesion of *E. sakazakii *to the epithelial and endothelial cells is mainly non-fimbrial based.

**Conclusion:**

Adherence experiments show heterogeneity within different *E. sakazakii *strains. In agreement with studies on *E. cloacae*, we found no relationship between the adhesive capacities in *E. sakazakii *and the eventual production of specific fimbriae. Further studies will have to be carried out in order to determine the adhesin(s) involved in the interaction of *E. sakazakii *with cells and to enhance knowledge of the pathogenesis of *E. sakazakii *infection.

## Background

*Enterobacter (E.) sakazakii *is considered a food borne pathogen that can cause severe illness and death in newborns, particularly in prematures or infants with weakened immune system [1,2]. Pathogenesis involves bacteraemia and/or sepsis, cerebro spinal fluid infection and meningitis, intracerebral infarctions, brain abscess or cyst formation, and has been associated with necrotizing enterocolitis (NEC) [3-7]. Infant mortality rate for *E. sakazakii *meningitis was reported to be 40 – 80 % [8-10] and up to 20% of the newborns develop serious neurological complications following infection [11]. NEC is characterized by the colonization of the gastrointestinal lumen and is the most common gastrointestinal emergency in newborns [12].

Although *E. sakazakii *has been isolated from a wide variety of foods, including cheese, meat, vegetables, grains, herbs, spices and ultra high heated milk [13-15], the bulk of research has been concentrated on the presence of the organism in milk-based powdered infant formula [12,16]. A positive correlation between NEC and oral formula feeding has been suggested [12,13].

To assess the pathogenicity of *E. sakazakii *for humans, evaluation of potential virulence factors is required. However, up to now little is known about the mechanisms of pathogenicity in *E. sakazakii*. A necessary state in the successful colonization, establishment and ultimately production of disease by microbial pathogens is the ability to adhere to host surfaces such as mucous membranes, gastric and intestinal epithelial or endothelial tissue [17,18]. Therefore, it is a common trait of microbial pathogens to express adherence factors responsible for recognizing and binding to specific receptor moieties of cells, thus enabling the bacteria to resist host strategies that would impede colonization. Specific adhesion to tissue cells is therefore considered an essential virulence factor for most bacterial pathogens [19].

Up to now, only two recent studies have described aspects of the interaction of *E. sakazakii *with cells. Pagotto et al. [20] examined in their work the pathogenesis and potential virulence factors of *E. sakazakii *clinical and food isolates exploring the suckling mouse assay and cell line experiments. Of 18 *E. sakazakii *strains (8 food strains, 9 clinical strains and 1 type strain (ATCC 29544) evaluated, four were found to be positive for enterotoxin production. Collando et al. [21] tested the ability of different bifidobacteria to inhibit different pathogen (*E. sakazakii *ATCC 29544 was included) adhesion and to displace pathogens previously adhering to cells in a human intestinal mucus model.

In this study, we investigated the potential of *E. sakazakii *strains from various origins to adhere to HEp-2 and Caco-2 cells, two well established and frequently used in-vitro models for studying interactions of bacteria with human cells. *E. sakazakii *can cause a highly lethal syndrome of bacteraemia and meningitis in neonates [3] with an extraordinarily high prevalence of developing a brain abscess [10,22]. Like *Citrobacter diversus *or *Citrobacter freundii*, *E. sakazakii *seems to have a tropism for the central nervous system, implying the organism's ability to breach the blood-brain barrier [22-24]. In an effort to understand the interaction of *E. sakazakii *with endothelial cells from the brain, the cell line HBMEC was used as the in-situ blood-brain barrier model [25].

Furthermore, the effects of bacterial culture conditions on the adherence behaviour were included in the study and an attempt was made to characterize the factors involved in adherence of *E. sakazakii *to mammalian cells. This is the first study providing data on the adherence ability in *E. sakazakii *strains.

## Results

**Figure 1:**
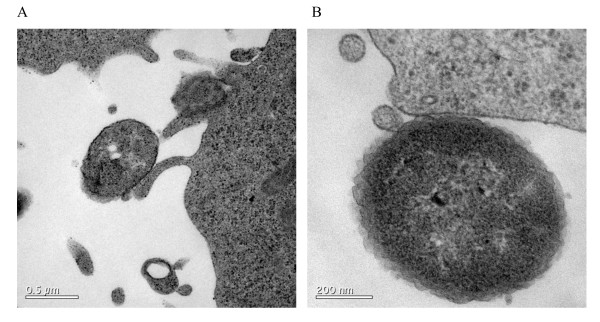
Transmission electron micrographs showing the adherence of *E. sakazakii *strain ES5 to Hep-2 cells. Bacterial cells appear in close contact with the mammalian cell membrane.

Results of the adhesion experiments of the 50 *E. sakazakii *strains on HEp-2, Caco-2 cells and HBMEC are summarized in Table 1. At least three replicates for all three cell lines were done. According to these results, adhesion was considered positive (+), when adhesion was observed in all the replicates, ambivalent (+/-), when adhesion was observed in one or two of the replicates and negative (-), when no adhesion was observed in any of the experiments. Briefly, positive adhesion was observed for 29 (58%) strains on HEp-2 cells, for 28 (56%) strains on Caco-2 cells and 25 (50%) strains on HBMEC. Adherence was confirmed by TEM observations revealing a tight association of bacteria to mammalian cell membranes (Figure 1). Sixteen (32%) strains showed ambivalent adhesion to all three cell lines and no adhesion was observed for 5 (10%) strains on HEp-2 cells, 6 (12%) strains on Caco-2 cells and 10 (18%) strains on HBMEC. Nineteen (36%) strains exhibited positive adhesion capacities to all three cell lines, 22 strains (44%) to both HEp-2 and Caco-2 cells and in four strains (8%) no adhesion was observed on any of the three cell lines. No difference was evident (P > 0.05) when adhesion behaviour was compared in view of the origin (human, environmental, food) of the *E. sakazakii *strains.

An adhesion index (< 1; >1; >100) describing the mean number of bacteria per mammalian cell after examination of 20 visual fields was determined (Table 1).

**Figure 2:**
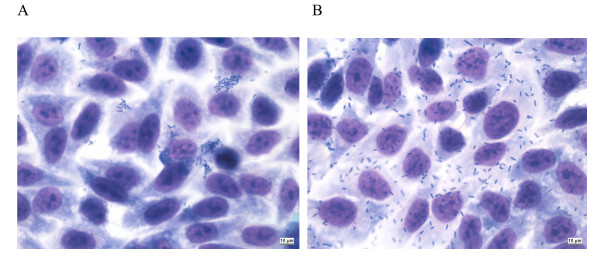
Light micrographs of *E. sakazakii *strain ES3 adhering in clusters (A) and strain ES6 showing diffuse adherence to HEp-2 cells (B). Methanol fixed and Giemsa stained.

Furthermore, three different adhesion patterns could be distinguished on all three cell lines: (i) association in clusters containing between 10 and >10^3 ^bacteria (ii) a diffuse adherence in which bacteria are distributed on the entire mammalian cell surface and (iii) a mixture of the two patterns (Table 1). Examples of the diffuse pattern and the adherence in clusters as observed on HEp-2 cells can be seen in Fig. 2.

### Factors influencing adherence

**Figure 3:**
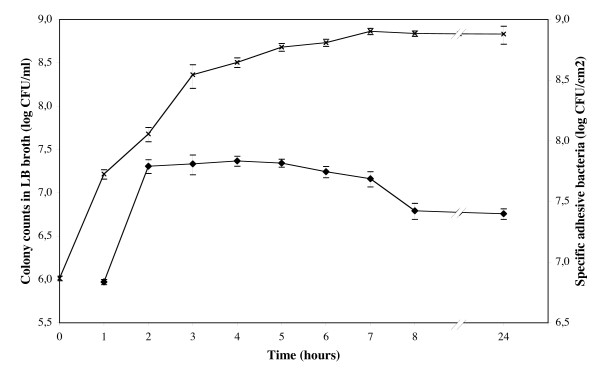
Effect of growth phase on adhesion of *E. sakazakii *strain ES5 to HEp-2 cells. The data represent the statistical means of three experiments. Symbols: *, colony counts in LB broth (log CFU/ml); •, number of specific adhesive bacteria (CFU per cm^2 ^of Hep-2 tissue culture).

Adhesion assays were performed with a constant number of viable, bacterial cells harvested at different stages of growth (Figure 3). Experimental results on the adhesion to HEp-2 cells indicated that the number of CFU per cm^2 ^recovered was at its maximum in the late exponential phase, i. e., with bacteria harvested after 4 h of culturing. Size of clusters increased when bacteria were collected during the exponential phase and the number of adherent bacteria increased by approx 10-fold. Additionally to the clusters, the appearance of an increasing number of diffuse adhering bacteria was observed when bacteria were collected during this phase. After reaching the stationary phase, adhesiveness of bacteria was reduced by half log and remained constant.

When control experiments were carried out using microplates without HEp-2 cells, bacterial adhesion to the plastic matrix was observed. The mean number of CFU recovered from the matrix was constant (2 × 10^6 ^CFU/cm^2^) along the growth curve.

Furthermore, adhesion of exponential (4 h) and stationary phase (16 h) cultures of *E. sakazakii *strain ES5 to HEp-2 cells was examined at different MOIs. The adhesion rate increased with the MOI, ranging from 4 × 10^5 ^(MOI 1:1) to 4 × 10^8 ^(MOI 1000:1) per cm^2 ^of cell monolayer.

The influence of incubation time on the adhesive behaviour was monitored at a constant MOI of 10:1. Our observations showed, that instant adherence (T = 0) occurred and reached its maximum at T = 180 min. These results indicate that the factor(s) necessary for bacterial adhesion is already present at a T of 0 min on both the host cell and at least in a fraction of the bacteria.

No adherence could be found for glutaraldehyde killed *E. sakazakii *cells at different MOIs. In addition, adhesion was not observed when infected HEp-2 cells were incubated at 4°C, indicating that metabolically active host cells were necessary for the adhesion process.

**Figure 4:**
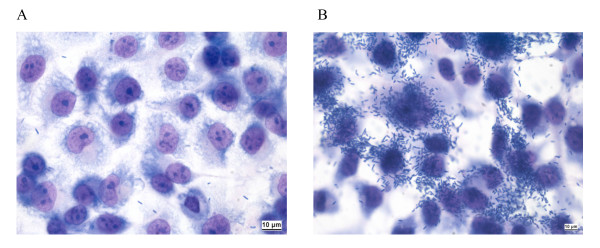
Light micrographs of *E. sakazakii *strain 3032 showing no adherence to HBMEC when grown in LB without blood (A) and (B) adhering in clusters after growth in LB supplemented with 20% sheep blood. Methanol fixed, stained with Giemsa.

Furthermore, *E. sakazakii *strain 3032, a blood culture isolate causing a fatal case of neonatal meningitis as well as human isolate ES5, were selected for growth experiments using 5 different media including TSB supplemented with sheep blood. No influence on adhesion was observed on the three cell lines, when the strains were grown in different media without blood. However, for *E. sakazakii *strain 3032 grown in TSB supplemented with blood, formation of clusters was observed on HBMEC cells (Figure 4). Moreover, the number of bacterial cells forming the aggregates increased with increasing concentrations of blood supplemented in the medium. Additionally, when the blood component in the medium reached 20%, appearance of diffuse adhering bacteria was observed.

In contrast, reduced adhesion capacity on HEp-2 cells was observed for strain ES5 when grown in medium supplemented with blood. Interestingly, for both strains adhesion to Caco-2 cells was not affected by growth in medium supplemented with blood.

Due to these unexpected results, all *E. sakazakii *strains included in the study were grown in blood supplemented TSB medium and adhesion on brain microvascular endothelial cells was examined. Significant changes were observed in the adhesive behaviour when some strains were grown in TSB supplemented with blood (Table 1).

### Characterization of adhesins

Pre-treatment of *E. sakazakii *strain ES5 bacteria with trypsin did not impair its adhesion to all three cell lines. Furthermore, addition of increasing amounts of mannose to the cell culture medium and bacterial growth medium did not reveal any reductions in adherence to HEp-2 cells. However, a reduction within the diffuse-adhering fraction of bacteria was observed with increasing mannose concentrations and when mannose concentrations reached 2%, no more diffuse adhering bacteria were observed. The bacterial fraction adhering in clusters, however, remained unchanged during the mannose experiments.

Hemagglutination assays on native and tannic acid treated horse erythrocytes revealed negative results for the two *E. sakazakii *strains tested under the conditions described. TEM on LB agar grown *E. sakazakii *colonies did not reveal the presence of fimbriae-like structures.

## Discussion

Two distinctive adherence patterns could be distinguished. Additionally, in a number of strains a mixture of those two patterns could be observed. In several studies, strains of opportunistic pathogens have been classified on the basis of their adherence pattern to tissue culture cells: aggregative patterns and diffuse adhesion [29,31,32]. The aggregative pattern of adhesion was previously recognized in *Klebsiella pneumoniae *strains associated with neonatal colitis and was found to be most prevalent phenotype in nosocomial strains [31]. However, for other organisms (e.g., *E. coli*), the association of adhesion patterns with different clinical syndromes is still controversial [33,34]. The results from our study showed that most *E. sakazakii *strains exhibited adhesion in clusters or the mixed pattern, regardless of their origin.

Furthermore, several parameters influencing adhesion such as bacterial growth phase, MOI and time of incubation were investigated. Our results can be summarized as follows: (i) When added at equal MOIs, a marginal difference was observed in adhesion when late logarithmic phase cells were used vs stationary-phase. (ii) The number of bacteria adhering to HEp-2 cells increased with increasing MOI and seemed to be limited only by the death of the host cell. Microscopic observations showed that both the size and the number of bacterial clusters increased with higher MOIs. The formation of clusters may take place by the replication of a few attached bacteria, and at higher MOIs, recruitment of bacteria present in the supernatant in the vicinity of the cell might occur. (iii) Adhesion occurs instantly at least for a fraction of the initial bacterial inoculum and increases in a logarithmic manner until an optimum is reached. (iv) Both host cells and bacteria have to be alive and metabolically active for adhesion.

The fact that we found reproducible formation of aggregative clusters for some strains, which showed no or ambivalent adherence under standard growth conditions, when grown in broth supplemented with sheep blood, is very interesting.

Badger and Kim [35] examined the influence of environmental growth conditions to the ability of *E. coli *K1 to invade HBMEC. Within this study, the influence of environmental pH on *E. coli *K1 invasion of HBMEC was discussed. Therefore, in our study, the pH was monitored during growth of bacteria in TSB media with and without (%) blood for *E. sakazakii *strain ES5 and 3032. During growth the pH decreased from 7.3–7.5 to 6.8–6.7 in both media, with and without blood. Thus, we concluded that the observed changes in the adhesive properties of bacteria grown in blood-supplemented media could not be explained by changes in pH. We therefore suggest that the adhesion behaviour of *E. sakazakii *strains might be influenced by other components available during growth in blood-supplemented media.

In view of the nature of the adhesive factors involved in the formation of clusters by *E. sakazakii *to HEp-2 cells it can be concluded that: (i) Trypsin digestion experiments did not impair the adhesion; (ii) Mannose sensitive type 1 fimbriae were not involved in cluster adhesion on Hep-2 cells. The observed decrease in diffuse-adhering bacteria could be explained by the possible presence of mannose sensitive type 1 fimbriae within this fraction, suggesting that adhesive behaviour is a multi-factorial process. Type 1 fimbriae are found in most strains of enterobacteria [36]. It is worth mentioning, that together with a very recent study [37], we published more than 89 kb of annotated *E. sakazakii *sequence data originating from a BAC library (TRCDSEMBL: AM075208) and among other information the presence of an operon putatively coding for type 1 fimbriae was predicted. However, to-date, there is no experimental data confirming the expression of these predicted genes; (iii) Haemagglutination experiments using native and tannic acid treated horse erythrocytes suggested that neither type 1 nor type 3 fimbriae were present under the conditions used. However, in order to confirm these results, experiments should be conducted using a broader range of erythrocytes from various animals [38]; (iv) TEM on LB agar grown *E. sakazakii *colonies did not reveal the presence of fimbriae-like structures; v) TEM observations revealed that the bacteria are tightly associated with HEp-2 cells, suggesting no involvement of fimbrial structures, as has been described for enterotoxigenic *E. coli *in the study by Darfeuille-Michaud [39] and (vi) the inclusion of several centrifugation steps (5 min at 3000 × g) prior to incubation with mammalian cells did not influence the adhesion capacity of the bacteria, suggesting that adhesion in *E. sakazakii *is not mediated by fragile fimbriae-like organellae.

## Conclusion

Adherence experiments show heterogeneity within different *E. sakazakii *strains. In agreement with studies on *E. cloacae *[31,40] we found no relationship between the adhesive capacities of *E. sakazakii *and the production of specific fimbriae. Further studies will have to be carried out in order to determine the adhesin(s) involved in the interaction of *E. sakazakii *with cells and to enhance knowledge of the pathogenesis of *E. sakazakii *infection.

## Methods

### Bacterial strains and culture conditions

In total 50 *E. sakazakii *strains from various origin (reference strains, human isolates, food isolates: fruit powder, milk powder, baby food and manufacturing factory environmental isolates) were used in the study (Table 1). For all adhesion assays, unless otherwise stated, bacterial cultures were prepared by inoculating Luria-Bertani Broth (LB) medium (Difco) with single colonies grown on blood agar plates and incubating them aerobically for 16 h at 37°C without shaking. For logarithmic subcultures the cultures were diluted 1:100 into fresh LB and incubated for 4 h at 37°C with shaking (150 rpm). For the growth phase experiments subcultures were obtained as described above except that the cultures were incubated up to 24 h.

### Cell cultures

HEp-2 and Caco-2 cells were grown (37°C, 5% CO_2_) in Minimum Essential Medium (MEM) (Gibco), supplemented with 10% heat inactivated fetal calf serum (Omnilab), 1% non-essential amino acids (Gibco), 2% GlutaMAX-I Supplement (Gibco) and 0.4 % gentamicin (Gibco). Culturing of HBMEC cells was performed as previously described [26]. For adhesion assays 10^5 ^cells per cm^2 ^were seeded onto circular glass cover slips (Sterilin, Barloworld Scientific) and incubated to confluency with cell culture medium without antibiotics for 48 hours (HEp-2, HBMEC) and 72 hours (Caco-2). Numbers of HEp-2, Caco-2 and HBMEC cells per well were determined by detaching the cells by Trypsin-EDTA treatment and counting them in a Neubauer chamber.

### Adhesion assays

Adhesion experiments on the *E. sakazakii *strain collection were performed following a modified method of the Center for Vaccine Development (University of Maryland), originally described by Cravioto et al. [27]. MOI (MOI; number of bacteria per number of mammalian cells) of 10:1 was added to the HEp-2, Caco-2 and HBMEC monolayers in 1 ml culture medium. Bacteria were allowed to adhere for 180 min at 37°C in 5 % CO_2_. *Escherichia coli *Transformax Epi100 was included as a non-adhering control. Aggregative adhering *E. coli *strain 1070 was included as a positive control. All experiments were performed in triplicates. After incubation, each well was rinsed five times with PBS, cell material was fixed by the addition of methanol for 10 min, stained with 10 % Giemsa stain (Sigma-Aldrich) and examined under a light transmission microscope at a magnification of × 1,000 (oil immersion).

Furthermore, adhesion of *E. sakazakii *strain ES5 to HEp-2 cells was visualized by TEM. HEp-2 cells were grown to 70–80% confluency on a 12 mm Polyester membrane with 0.4 mm pore size (Costar Transwell, Corning). The cell monolayers were washed twice with PBS (Gibco) and once with fresh cell culture medium and 10 ml stationary phase bacterial culture (MOI 10:1) was added to the membrane at the bottom of the wells. The plate was centrifuged for 10 min at 1,500 × g at room temperature and incubated for 90 min at 37°C in 5% CO_2_. The membrane was washed, fixed and embedded according to standard procedures. Ultra-thin sections (80 nm) were investigated under the electron microscope (Philips CM10).

### Factors influencing adherence

Unless otherwise stated, confluent monolayers of HEp-2 cells and *E. sakazakii *strain ES5 at a constant MOI of 10:1 were used for the experiments. All experiments were performed in duplicates and evaluated by light microscopy.

In order to quantify adherent bacteria, 0.2 ml of 0.1% Triton X-100 (Sigma) was added to each well containing mammalian cells for 10 min. After addition of 0.8 ml of saline (0.85% NaCl) released bacteria were quantified on LB agar plates. Control wells without mammalian cells were prepared in a similar manner in order to quantify non-specific bacterial adherence to the plastic well. Specific adhesion to HEp-2 cells was expressed as the total number of CFU minus the number of CFU adherent to wells without mammalian cells per cm^2 ^of tissue culture well.

#### (i) Growth phase

Subcultures were obtained and incubated in a shaker up to 24 h. The 1 h-, 2 h-, 3 h-, 4 h-, 5 h-, 6 h-, 7 h-, 8 h-, and 24 h- subcultures were harvested by centrifugation, resuspended and optical density at 620 nm was adjusted in order to ensure the use of the same number of bacteria for each experiment. An aliquot of the diluted subculture (100 ml = 4 x10^8 ^bacteria) was added to each tissue culture plate well. Bacterial cells and monolayers were incubated for 120 min.

#### (ii) MOI

HEp-2 monolayers were treated as described in the growth phase experiments and bacterial cultures of stationary and exponentially grown *E. sakazakii *strain ES5 were added at different MOIs of 1:1, 5:1, 10:1, 50:1, 100:1, and 1000:1. Bacterial cells and monolayers were incubated for 90 min.

#### (iii) Time of incubation

HEp-2 monolayers were infected with a constant MOI of 10:1 After centrifugation, bacterial cells and monolayers were incubated for 0, 10, 20, 30, 40, 50, 60, 90, 120, 150, 180, 210, 360, and 540 min.

#### (iv) Viability of bacteria

Experiments were performed following the method described by Kusters et al. [28]. Stationary phase cultures of *E. sakazakii *strain ES5 were collected after centrifugation (5 min at 3,000 × g) and suspended in prewarmed (37°C) DMEM (Oxoid) without supplements. The bacterial pellet was resuspended in cold 2.5% glutaraldehyde in PBS and incubated for 1 h at 4°C. Following centrifugation (5 min at 3,000 × g), the glutaraldehyde-PBS was removed and the bacteria were resuspended in DMEM and incubated at 4°C for 30 min. After two additional 30-min DMEM washes, the bacteria were resuspended and diluted in prewarmed DMEM as described above. From this suspension, MOI of 10:1 to 100:1 were added to confluent HEp-2 cells prepared as described above and incubated for 90 min. As a positive control, the same experiment was performed without 2.5% glutaraldehyde.

#### (v) Temperature dependence

In order to determine the contribution of metabolism of the host cells and the bacteria to the adhesion process, 9 *E. sakazakii *strains (759/03, 954/03, ES4, ES5, ES6, ES14, FSM265, FSM271, FSM423) from different origin were selected and incubated with HEp-2 cells at 37 and 4°C for 90 min.

#### (vi) Growth medium

In the first part of the experiment, *E. sakazakii *strains ES5 and 3032 were selected and grown in different media for 16 h at 37°C with moderate shaking and adhesive abilities were examined on HEp-2, Caco-2 and HBMEC respectively. The following growth media were used: LB, TSB, peptone buffered water (Oxoid), BHI as well as TSB supplemented with 5, 10, 20, 40% defibrinated sheep blood (Oxoid). Monolayers were infected and incubated for 180 min. In the second part of the experiment, all *E. sakazakii *strains included in the study were grown in TSB supplemented with 20% defibrinated sheep blood and adhesion to HBMEC was examined.

### Characterization of adhesins

#### Trypsin

Experiments were performed by a modified method described by Favre-Bonte et al. [29] on all three cell lines and using *E. sakazakii *strain ES5. Prior to infection, LB grown bacterial culture was treated with 2 mg/ml Trypsin for 1 h. Bacteria were added and monolayers were incubated for 180 min.

#### Mannose

HEp-2 cells and *E. sakazakii *strain ES5 bacteria were treated and incubated as described above, except that 0.5, 1, and 2 % mannose was added to both bacterial culture medium (TSB) and mammalian cell culture medium.

#### Hemagglutination

Experiments with *E. sakazakii *strains ES5 and 3032 were performed according to the method described by Old [30]. Hemagglutination was assessed after incubation of 20 min at room temperature. *Salmonella *Enteritidis and *Klebsiella pneumoniae *were used as positive controls for hemagglutination to fresh and tannic acid-treated horse erythrocytes, respectively. Saline was served as a negative control.

#### Visualization of fimbriae

For visualization of possible fimbriae by TEM, *E. sakazakii *strain ES5 was grown on LB agar plates and colony material was suspended in 12 × Tris/acetate EDTA electrophoresis buffer (TAE), pH 7.5, centrifuged at 2.500 × g for 10 min and resuspended in 100 mMTris/HCl, 7.5. Bacteria were then adsorbed for 5 min to 7 nm thick glow discharged carbon coated parlodion films mounted on 300-mesh per inch copper grids, stained with 0.4% phosphotungstic acid, pH 6.6, for 1 min, air-dried and immediatly examined in a Philips CM12 electron microscope (Philips, Eindhoven, The Netherlands) at an acceleration voltage of 100 kV. Images were recorded with a slow scan CCD camera (Gatan Inc. Pleasanton, California, USA).

## Authors' contributions

JPM and NB carried out the cell adhesion experiments. AP and PW participated in the TEM experiments. RS conceived of the study. RS, KSK and AL participated in its design and coordination and helped to draft the manuscript. All authors read and approved the final manuscript.

## Figures and Tables

**Table 1:** Origin and adhesion capacities of 50 *E. sakazakii *strains on HEp-2, Caco-2, HBMEC and HBMEC after growing in blood-supplemented medium.

*E. sakazakii*		Adhesion properties											
		
		Hep-2			Caco-2			HBMEC			HBMEC (blood)		
ATCC 51329		+	**	C/D	+/-	*	C/D	+	**	C	+	**	C
ATCC 29004		+	*	C/D	+	**	C/D	-			+/-	*	C
DSM 4485		+/-	*	C/D	+	*	C/D	+/-	*	C	+	*	C
													
Es 3	Human^1^	+	**	C	+	*	C	+	**	C	+	**	C
Es 4	Human^1^	+	**	C/D	+	*	C/D	+	*	C/D	+	**	C/D
Es 5	Human^1^	+	**	C/D	+	**	C/D	+	**	C	+	**	C
Es 6	Human^1^	+	**	C/D	+	***	D	+	*	C/D	+	*	C
Es 7	Human^2^	+	**	C	+	*	C	+	**	C	+	***	C/D
Es 10	Human^1^	+/-	*	C/D	+/-	*	C/D	+/-	*	C	+/-	*	C
Es 11	Human^1^	+/-	*	C/D	+	*	C	+/-	*	C/D	-		
Es 12	Human^1^	+	**	C/D	+/-	*	C	+/-	*	C	+	*	C
Es 13	Human^2^	+	**	C	+/-	*	C	+	**	C	+	**	C
Es 14	Human^1^	+	**	C/D	+	*	C/D	+/-	*	C	+	*	C/D
Vo7/24922	Human^1^	+/-	*	C	+	*	C	+/-	*	C/D	+	*	C/D
3032/05	Human^3^	+	**	C/D	+/-	*	C	-			+	**	C
FSM 477	Human^1^	+/-	*	C	+/-	*	C	-			+/-	*	C
													
732/03	Fruit powder	+/-	*	C/D	+	*	C	+	**	C	+	*	C
858/03	Fruit powder	+	**	C/D	+	*	C	+	*	C	+	*	C
236/04	Fruit powder	+/-	*	D	-			+	*	C/D	-		
759/03	Fruit powder	+/-	*	C/D	+	*	C	+/-	*	C/D	+	*	C
954/03	Fruit powder	+	**	C/D	+	*	C	+	**	C/D	+	**	C
130/03	Fruit powder	+	**	C/D	+	*	C	+	**	C/D	+	**	C
974/02	Fruit powder	+	**	C/D	+	*	C	+	**	C	+	*	C
1103/03	Fruit powder	+	**	C/D	+	*	C	+	**	C	+	**	C
324/04	Fruit powder	+/-	*	D	+	*	D	+/-	*	D	+/-	*	C
966/04	Fruit powder	+/-	*	C/D	+/-	*	C	+/-	*	C	+	*	C
1084/04	Fruit powder	+	**	C	+	*	C	+	**	C/D	+	**	C/D
1154/04	Fruit powder	-			-			-			-		
1155/04	Fruit powder	+/-	*	C	+/-	*	C	-			-		
1156/04	Fruit powder	+/-	*	C/D	+/-	*	C	+/-	*	D	+/-	*	C
1386/04	Fruit powder	+	**	C/D	+	*	C/D	+	**	C	+	**	C
													
FSM 33	Milk powder	+/-	*	C/D	+/-	*	C	-			-		
FSM 265	Milk powder	+	***	C/D	+	*	C/D	+	*	C/D	+/-	*	C/D
FSM 328	Baby food	-			-			-			-		
FSM 393	Baby food	-			-			-			-		
													
FSM 266	Environment	+	**	C/D	+	*	C/D	+	*	C/D	+	***	C/D
FSM 269	Environment	-			-			-			-		
FSM 271	Environment	+	***	C/D	+	**	C/D	+	**	C/D	+	**	C
FSM 286	Environment	+	***	C/D	+	**	C/D	+	**	C/D	+	**	C/D
FSM 290	Environment	+	***	C/D	+	*	C/D	+/-	*	C	+	*	
FSM 298	Environment	+/-	*	C/D	+/-	*	C	+	**	C	+	**	C
FSM 311	Environment	+/-	*	D	+/-	*	C/D	-			-		C
FSM 318	Environment	+	**	C/D	+/-	*	C	+/-	*	C	-		
FSM 322	Environment	+	**	C/D	+	*	C	+	**	C/D	+	***	C/D
FSM 323	Environment	+	**	C/D	+	*	C	+	**	C	+	**	C/D
FSM 423	Environment	+	***	C/D	+	**	C/D	+	**	C/D	+	***	C/D
FSM 464	Environment	+	***	C	+/-	*	C	+/-	*	C/D	+/-	**	C
FSM 465	Environment	+/-	**	C	+/-	*	C	+	**	C/D	+	**	C/D
FSM 468	Environment	+	**	C/D	+/-	*	C	+/-	*	C/D	+	**	C
FSM 469	Environment	-			-			+/-	*	C/D	+/-	*	C/D

+ positive adhesion, +/- ambiguous adhesion in parallel experiments, - no adhesion

* adhesion index of < 1, ** adhesion index of >1, ***adhesion index of > 100

C: adhesion in clusters, D: diffuse adhesion, C/D mixed pattern

Human^1 ^: source unknown, Human^2 ^: blood culture, Human^3 ^: blood culture, strain responsible for neonatal meningitis and death of 2 newborns in Zurich 2005
